# The Relevance of Discovering and Recovering the Biodiversity of Apulian Almond Germplasm by Means of Molecular and Phenotypic Markers

**DOI:** 10.3390/plants11040574

**Published:** 2022-02-21

**Authors:** Michele Antonio Savoia, Loredana Del Faro, Pasquale Venerito, Liliana Gaeta, Marino Palasciano, Cinzia Montemurro, Wilma Sabetta

**Affiliations:** 1 Department of Soil, Plant and Food Sciences, University of Bari Aldo Moro, Via Amendola 165/A, 70126 Bari, Italy; michele.savoia@uniba.it (M.A.S.); marino.palasciano@uniba.it (M.P.); cinzia.montemurro@uniba.it (C.M.); 2 CRSFA-Centro Ricerca, Sperimentazione e Formazione in Agricoltura “Basile Caramia”, Via Cisternino 281, 70010 Locorotondo, Italy; loredana.delfaro@posta.istruzione.it (L.D.F.); pasqualevenerito@crsfa.it (P.V.); 3 Council for Agricultural Research and Economics-Agriculture and Environment Research Centre (CREA-AA), Via Celso Ulpiani 5, 70125 Bari, Italy; liliana.gaeta@crea.gov.it; 4 Spin Off Sinagri s.r.l., University of Bari Aldo Moro, Via Amendola 165/A, 70126 Bari, Italy; 5 Institute for Sustainable Plant Protection–Support Unit Bari, National Research Council (IPSP-CNR), Via Amendola 165/A, 70126 Bari, Italy; 6 Institute of Biosciences and BioResources, National Research Council (IBBR-CNR), Via Amendola 165/A, 70126 Bari, Italy

**Keywords:** *Prunus dulcis*, microsatellites, phenotypic descriptors, local biodiversity safeguard

## Abstract

Almond cultivation has great traditional and economic relevance in Southern Italy, especially in the Apulia region, where almond trees feature an ample and ancient varietal richness. To contrast the loss of plant genetic erosion and to safeguard the available bioresources, as well as to reinforce the local production, the regional Re.Ge.Fru.P. project aimed to re-evaluate, identify, and characterize the Apulian almond germplasm that is still uncharacterized and not jet studied using a dual (genetic and morphological) approach. Collection was conducted in the regional territory of 187 among the most widespread and minor or marginalized genotypes that were molecularly fingerprinted by means of 18 nuclear microsatellites (simple sequence repeats, SSRs). The high number of scored alleles reflected the great level of diversification within the Apulian germplasm, as also confirmed by neighbor joining and structure analysis, that clearly distinguished different genotype clusters. The phenotypic characterization using 17 morphological and phenological descriptors mirrored the genetic results, revealing a high degree of variability. The morphological traits with the best discriminatory ability were nut ventral suture, shell softness and shape and petal color. This work emphasizes the importance of recovering the genetic variability of Apulian almond germplasm, and the need to promote added value and enhance the local agri-food economy.

## 1. Introduction

Almond (*Prunus dulcis* Mill. D.A. Webb., syn. *Prunus amygdalus* Batsch, syn. *Amygdalus communis* L.) is probably the oldest nut tree crop to be domesticated; indeed, the first signs of its cultivation appeared in literature as early as 3000 B.C. [1]. Native to Central Asia [2], it spread over several centuries to the West, mainly toward the Mediterranean basin, where it was widely cultivated and then spread further, all over the world, spontaneously or by human seed dissemination. Thus, the Mediterranean basin can be seen as a second centre of domestication of this species. Today, almond is considered the most important nut tree crop in the world, the USA, Spain and Australia being the primary producers [3]. In Italy, it is cultivated on a large scale in the South, particularly in the regions of Sicily and Apulia, accounting for 96% of the national production [4]. A smaller production, mainly based on local varieties, occurs in other Italian regions such as Sardinia, Calabria, Abruzzo and Basilicata.

Despite a high genetic similarity to other *Prunus* species, almond has fleshless fruits and an edible kernel, rather than edible fruits. The importance of this crop has increased in the human diet thanks to the discovery of its health benefits and nutritional value, that have consequently intensified marketing of this product worldwide. As a good source of natural antioxidants with bioactive properties, almond is widely used in food as well as in pharmaceutical and cosmetic industries, which require kernels with a high-quality oil content [5]. Clinical studies have demonstrated that almond consumption is associated with a reduction of cardiovascular risk and the induction of favorable plasma lipid profiles, mainly related to its oil composition [6,7,8].

At the end of the 20th century, the Italian almond production underwent a sharp decrease, that had considerable economic consequences on the national market [9]. This reduction was attributed to several causes, such as low or variable annual yield, inefficient orchard management and maintenance practices [10,11], and the spread of diseases [12]. These problems, together with unfavorable import and export conditions, caused the abandonment of many local cultivars and hence a high risk of genetic erosion. To reverse this process and avoid the extinction of autochthonous almond trees over the years, a classification of the local genotypes has been made, and the creation of almond germplasm collections with a wide genetic diversity started in Italy [9,13,14,15,16,17,18].

Since then, the interest in traditional and autochthonous varieties has been growing continuously, especially now that the great importance worldwide of safeguarding and protection of biodiversity has been recognized. Ancient and rare plant germplasm could offer a precious bioresource of agronomically important traits and genes, that could potentially be useful to cope with climate changes and to adapt to new conditions. Thus, in 2013, the Apulia region financed an integrated project named Re.Ge.FRU.P. (Apulian Fruit Germplasm Recovery) with the aim of recovering the biodiversity of several Apulian fruit tree species, including almond, by means of various different actions, including historical investigation and cataloguing, morphological and genetic characterizations as well as ex situ conservation of local germplasm. In this context, robust methods to discriminate nonidentical individuals were essential in order to pursue some of the specified goals. The traditional methods for cultivar characterization and fruit tree identification, based on phenotypic observations and morphological descriptions, are usually coupled with the more accurate DNA-based methods. Together, they act as an extremely useful tool in breeding programs, providing a fast and indisputable fingerprint of genotypes and for the assessment of genetic relationships [19].

Several studies were conducted to characterize different almond germplasm and evaluate the level of genetic variability within populations, using different molecular markers, such as RAPDs [20,21], AFLPs [22] and SNPs [23,24]. In particular, microsatellites (SSRs), which are often the markers of choice thanks to their numerous advantageous features (hypervariability, codominance, multiallelic nature, high reproducibility and extensive genome coverage) [25,26], have been widely used for the evaluation of almond genetic diversity [27,28,29,30], germplasm management [31,32], genome mapping and syntheny analysis [33,34].

Given the economic and cultural importance of almond cultivation in the Apulia region, and the high interest in contrasting plant genetic erosion, the main aims of this work were to study the genetic structure of Apulian almond germplasm and to enrich the collection of already available genetic resources, in particular safeguarding ancient and local genotypes and assessing their potential for breeding purposes.

## 2. Results and Discussion

### 2.1. SSR Polymorphism

In order to evaluate the genetic diversity of the 187 Apulian almond genotypes, an in-depth molecular characterization was performed using 18 SSR markers, chosen on the basis of their dispersal map location. Capillary electrophoresis analysis produced clear genotyping profiles for all the examined loci, yielding good and reproducible amplification products within the expected allele-size ranges (Table 1). A total number of 298 alleles (Na) was obtained, with an average value of 16.5 alleles per locus, ranging from a minimum of 5 for BPPCT014 to a maximum of 22 alleles for UDP96005 and BPPCT025. This mean value was slightly lower than that observed by [35] (18.0) and by [29,36] (17.2 and 18.7, respectively) in their studies of almond genetic diversity, but it was perfectly in line with what was reported by [37] (16.8) for the characterization of an Iranian almond collection.

The mean of the effective alleles (Ne) was 6.10, ranging from 2.23 for BPPCT014 to 11.00 for CPDCT045, slightly lower as compared to two other reports [29,37]. For each microsatellite, the PIC (Polymorphism Information Content) value, i.e., marker informativeness and richness in terms of number of allelic forms [38], was calculated. In general, the molecular analysis revealed a high degree of polymorphism for all the loci considered, as the PIC values were always greater than 0.681, the mean being 0.783. The only exception was the BPPCT014 marker, whose PIC value was 0.453, thus resulting the least polymorphic microsatellite. In accordance with these findings, all SSRs except BPPCT014 harbored at least two private alleles (allelic frequency < 1%), the greatest number (9 private alleles) being observed for BPPCT025 and UDP96005. Comparable PIC values have been observed in other research works [21,37,39], where the microsatellites used had mean discrimination powers of 0.79, 0.81 and 0.80, respectively. Interestingly, rather higher PIC values (0.85 and 0.92) were reported in the studies carried out by [35,40].

The observed Heterozygosity (Ho) ranged from 0.335 (UDP98409) to 0.935 (CPDCT045) with a mean value of 0.711, while the expected Heterozygosity (He) varied between 0.553 for BPPCT014 to 0.909 for CPDCT045, with an average measure of 0.807 (Table 1). This difference determined positive fixation index (F) values (mean 0.116) for almost all markers, as a measure of genetic diversity; only two microsatellites, i.e., BPPCT014 and CPDCT045, were exceptions, since their F index value was negative (−0.006 and −0.028, respectively) (Table 1). The average Ho value calculated in our study was similar to those obtained by [29] (Ho = 0.72), [35] (Ho = 0.71) and [39] (Ho = 0.73), resulting quite a lot higher than the values reported by several other authors [21,34,36,37,41]. In general, in all these research studies, the He values were higher than Ho, thus determining positive fixation indexes for most of the microsatellites used. The great heterozygosity of almond cultivars is in line with the mating system of this species that is normally self-sterile and out-crossing [41]. Thus, the high number of detected alleles and the heterozygosity value reflected the ability of SSR markers to provide a unique genetic profile for each individual genotype. The presence of null alleles (Nu) was also detected in our study. A null allele frequency greater than 0.2 is usually considered the threshold above which a significant underestimation of the expected heterozygosity due to null alleles is possible [42,43]. Nu values higher than 0.20 were obtained for 4 of the 18 microsatellites used, namely the BPPCT001 (0.3486), CPSCT018 (0.2918), UDP98409 (0.3725) and UDP98412 (0.3803) loci (Table 1). For this reason, these loci were not considered in further analyses.

Moreover, Shannon’s information Index (I) ranged from 0.926 (BPPCT014) to 2.608 (CPDCT045), with an average value of 2.052, indicating a high level of genetic diversity in the collection studied (Table 1).

**Table 1:** Genetic diversity indices estimated for the considered SSR loci in the Apulian almond collection. For each locus, the allele size range (basepair, bp), the number of detected alleles (Na), the effective number of alleles (Ne), the observed (Ho) and expected (He) heterozygosity, the fixation index (F), Shannon’s index (I), the PIC value and the frequency of null alleles (Nu) are reported. The presence of null alleles is highlighted in bold.

SSR Locus	Size	Na	Ne	Ho	He	F	I	PIC	Nu
BPPCT001	101–159	21.0	5.627	0.408	0.822	0.504	2.197	0.805	**0.3486**
BPPCT007	119–159	15.0	6.704	0.754	0.851	0.114	2.091	0.834	0.0573
BPPCT010	122–162	18.0	8.193	0.832	0.878	0.052	2.336	0.866	0.0229
BPPCT014	178–198	5.0	2.236	0.556	0.553	−0.006	0.926	0.453	−0.0060
BPPCT025	149–197	22.0	6.095	0.780	0.836	0.067	2.226	0.820	0.0338
CPDCT025	156–198	14.0	4.576	0.747	0.781	0.044	1.954	0.764	0.0192
CPDCT042	160–222	21.0	7.227	0.819	0.862	0.049	2.319	0.849	0.0262
CPDCT045	126–176	20.0	11.001	0.935	0.909	−0.028	2.608	0.902	−0.0154
CPPCT006	172–206	18.0	8.385	0.860	0.881	0.023	2.367	0.870	0.0108
CPPCT033	127–165	13.0	4.575	0.766	0.781	0.019	1.814	0.750	0.0069
CPSCT012	140–186	17.0	3.495	0.572	0.714	0.198	1.835	0.699	0.1181
CPSCT018	145–177	14.0	7.086	0.472	0.859	0.451	2.169	0.843	**0.2918**
EPPCU5176	106–134	16.0	4.168	0.746	0.760	0.019	1.838	0.730	0.0075
PCHGMS1	180–222	14.0	7.067	0.797	0.859	0.072	2.135	0.843	0.0364
UDP96003	87–137	14.0	3.488	0.713	0.713	0.000	1.678	0.681	−0.0011
UDP96005	126–196	22.0	6.535	0.759	0.847	0.103	2.337	0.835	0.0581
UDP98409	120–170	19.0	3.553	0.335	0.719	0.534	1.930	0.706	**0.3725**
UDP98412	94–134	15.0	6.231	0.376	0.840	0.552	2.177	0.824	**0.3803**
*Mean value*	*-*	*16.5*	*6.105*	*0.711*	*0.807*	*0.116*	*2.052*	*0.783*	*0.0709*

The allelic similarity among the Apulian almond genotypes analyzed was also calculated by means of LRM estimation (pairwise relatedness), setting 0.5 as maximum value for identical genetic profiles (cases of synonymy). A complete genetic identity was observed for the cultivars “Mollesca di Ruvo”, “Troito”, “Tuono” and “Stilla” (Table 2). The genetic similarity of “Tuono” with “Troito”, as well as with other Italian and foreign cultivars such as “Supernova” [44], “Moncajo” and “Laurenne” [45], has been previously reported by other authors and is probably due to the extensive use of this cultivar in breeding programs, as they are good sources of self-compatibility [24,46,47]. Therefore, as “Mollesca di Ruvo” and “Stilla” also showed a high genetic similarity with “Tuono”, we hypothesized that, together with “Troito”, they could be Tuono-related cultivars, probably assigned different names due to their morphological diversity.

Most of the examined genotypes had LRM values between 0.4 and 0.5, thus resulting unique individuals with no case of synonymy (Table 2). High LMR values were found for the couples “Bianchetta-Lunghina”, “Del lago-Rachele tenera” and “Riviezzo Grosso-Riviezzo Piccolo” and for the groups “Bianchetta-Lunghina-Secolare Cotogni” and “Mollesca di Ruvo-Mollese grossa_2-Tuono-Troito-Stilla-Piangente”.

To our knowledge, the evaluation of the LRM parameter has never previously been reported in other studies about almond genetic diversity.

**Table 2:** List of pairwise relatedness based on the LRM estimator.

Genotypes with LRM = 0.5		
Mollesca di Ruvo	Stilla	0.50
Mollesca di Ruvo	Troito	0.50
Stilla	Troito	0.50
Mollesca di Ruvo	Tuono	0.50
Stilla	Tuono	0.50
Troito	Tuono	0.50
**Genotypes with 0.4 < LRM < 0.5**		
Bianchetta	Lunghina	0.49
Del lago	Rachele tenera	0.48
Riviezzo Grosso	Riviezzo Piccolo	0.47
San Giuseppe_2	Troia	0.46
Mollesca di Ruvo	Mollese grossa_2	0.45
Mollese grossa_2	Stilla	0.45
Mollese grossa_2	Troito	0.45
Mollese grossa_2	Tuono	0.45
Bianchetta	Secolare Cotogni	0.45
Ficanera	Mollese di canneto	0.44
Ciavea	San Michele strada	0.44
Chino	Rachele tenera	0.44
Don Carlo	San Michele strada	0.44
Lunghina	Secolare Cotogni	0.44
Montranese	Zin Zin	0.43
Pilella	Scquicciarina	0.43
Chino	Del lago	0.43
Gianfreda	Riviezzo Grosso	0.42
Mollesca di Ruvo	Piangente	0.42
Piangente	Stilla	0.42
Piangente	Troito	0.42
Piangente	Tuono	0.42
Pettolecchia	Strappasacco	0.42
Gianfreda	Riviezzo Piccolo	0.41
Portone Gioia	San Michele strada	0.41
Ciavea	Zin Zin	0.41
Ferrante	Sciddiata Nisi	0.40

### 2.2. Genetic Characterization

**Figure 1:**
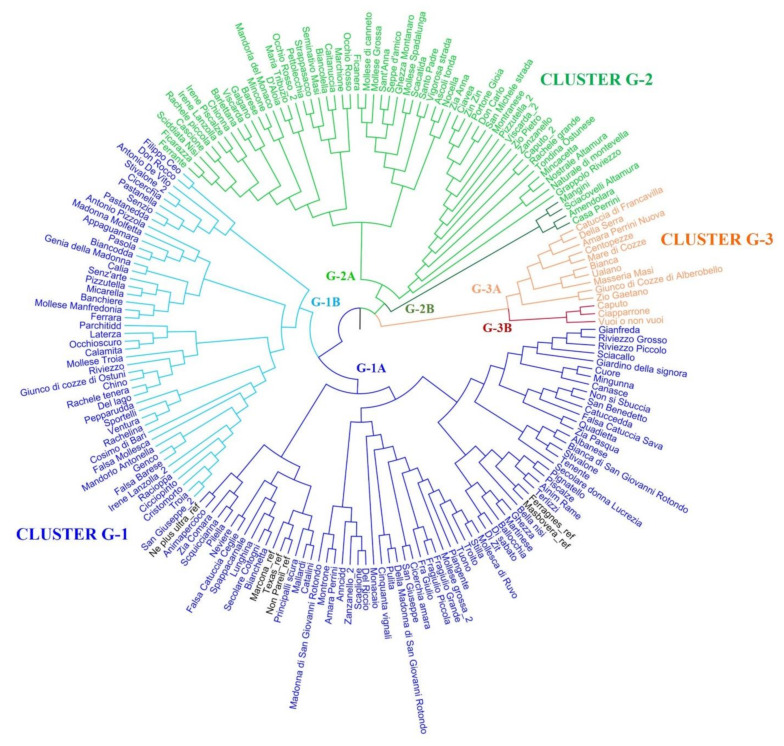
Dendrogram of the Apulian almond population based on genetic distance. Genotypes names are colored according to the main clusters they belong to (blue for G-1, green for G-2, and orange for G-3). Reference cultivars are colored black.

The neighbor-joining analysis allowed us to obtain a phylogenetic tree and to subdivide the Apulian almond population into three main clusters: G-1, G-2, and G-3 (Figure 1). The great majority of genotypes was included in cluster G-1, featuring 116 individuals, while 58 genotypes were grouped together in cluster G-2; cluster G-3 resulted the least abundant, including only 13 genotypes. Moreover, all these major clusters contained at least two recognizable subgroups, that we denominated G-1A and G-1B, G-2A and G-2B, and G-3A and G-3B (Figure 1). All the commercial almond varieties used as references were included in the G-1 group, in three different specific branches of the subcluster G-1A. In particular, the two Spanish cultivars, “Masbovera” and “Marcona”, were found to be moderately distant from one another, instead resulting genetically closer to the French cultivar “Ferragnes” and to the American cultivars “Texas” and “Non Pareil”, respectively. These findings are in agreement with what was observed by [35], since in that study, too, “Masbovera” skipped clustering with the American cultivars and was grouped together with some Sicilian genotypes. The third American cultivar “Ne plus ultra”, here used as reference, fell into a different branch of clade G-1A, moderately close to the other Americans, similarly to what was reported by [48]. Some Apulian genotypes appeared to be genetically more closely related to the Spanish and the American commercial varieties than others, probably as a consequence of the gene flow between commercial varieties and local materials. In general, the Italian almond germplasm has proven to show a high level of mixed ancestry among western areas of the Mediterranean basin, mainly due to human migration along the reconstructed maps of ancestral trade routes [49].

The phylogenetic clusterization of the Apulian genotypes was generally in line with the LRM values obtained, since cultivars with a high allelic similarity were found to belong to the same branch of the tree (Figure 1). For example, the close genetic distance between “Mollesca di Ruvo” and “Stilla” confirmed that they share the same genetic profile, while “Troito” and “Tuono” seemed to exhibit some genetic differences despite their LRM = 0.5. Strong genetic relationships were also confirmed for “Bianchetta”, “Lunghina” and “Secolare Cotogni” and for “Rachele tenera” and “Del lago”. With the exception of “Rachelina”, that was in the same subgroup G-1B together with “Rachele tenera”, the other genotypes including the term “Rachele” in their name, i.e., “Rachele grande” and “Rachele piccola”, did not show such high genetic similarity, since they fell into two different branches of cluster G-2A. The cultivar “Riviezzo Grosso”, whose LRM value was equal to 0.47, with “Riviezzo Piccolo”, appeared closely related to the cultivar “Gianfreda” (subgroup G-1A). Additionally, both “Riviezzo Grosso” and “Riviezzo Piccolo” were quite distant from the cultivar “Riviezzo”, that was instead included in subgroup G-1B together with “Rachele tenera”, as also observed by [48].

One group of genotypes (“Ciavea”, “Zin zin”, “Portone Gioia”, “Don Carlo”, “San Michele strada” and “Montranese”), with moderately high LRM values ranging from 0.41 to 0.44, together formed a branch of the tree in cluster G-2A. Interestingly, only four genotypes constituted cluster G-2B, namely the cultivars “Mangini”, “Sciacovelli Altamura”, “Amendolara” and “Casa Perrini”.

Some cases of homonymy were discovered, since genotypes with the same name but genetically polymorphic were clearly distinguished (Figure 1). Among them, the cultivars “Caputo” and “Caputo_2”, “Irene Lanzolla” and “Irene Lanzolla_2”, “Pizzutella” and “Pizzutella_2”, “San Giuseppe” and “San Giuseppe_2”, “Stivalone” and “Stivalone_2”, “Viscarda” and “Viscarda_2”, and finally “Zanzanello_1” and “Zanzanello_2” are noteworthy, as the components of each pair belonged to clearly different subgroups or to different main clades in some cases, thus resulting phylogenetically distant. Moreover, the case of the two homonymous cultivars “Mollese Grossa” and “Mollese Grossa_2” was also resolved because surprisingly, they were quite distant from one another and clustered in different clades, G-2A and G-1A, respectively. By contrast, “Mollese Grossa” was close to “Mollese di Canneto” and belonged to the same branch of the cultivar “Mollese Spadalunga”, while “Mollese Grossa_2” was grouped together with “Tuono-Troito-Stilla”. Despite the name similarity, other two genotypes named “Mollese Troia” and “Mollese Manfredonia” skipped these groupings and fell into another different cluster, i.e., the subgroup G-1B.

### 2.3. Population Structure

**Figure 2:**
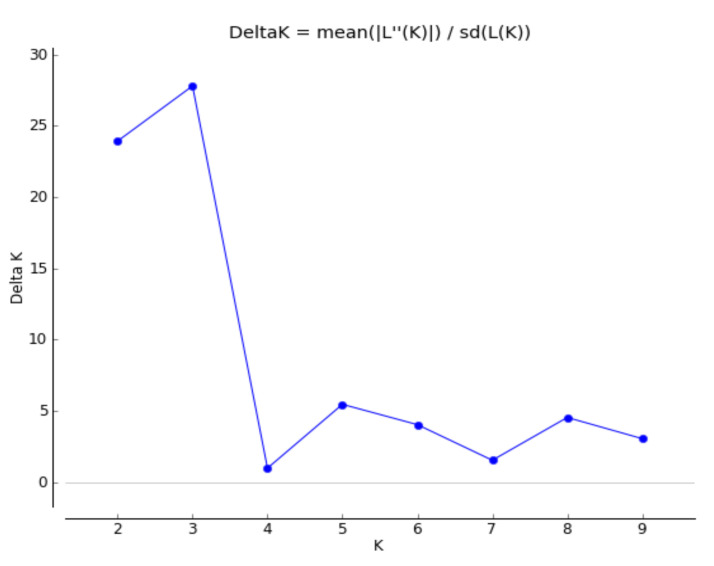
Estimation of the optimum number of clusters for the Apulian almond population according to Evanno’s method. For each K value, the DeltaK is reported, thus indicating that K = 3 is the uppermost probable number of genetically homogenous groups in the collection analyzed.

**Figure 3:**
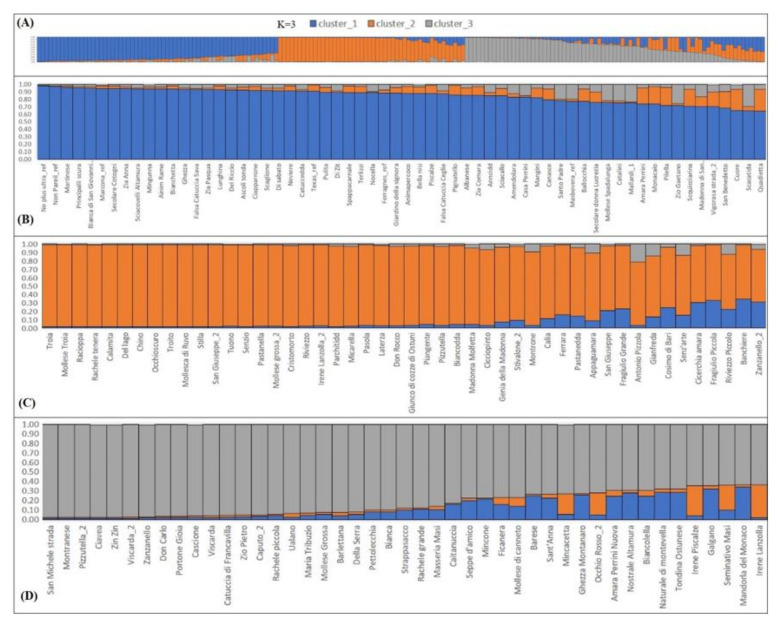
(**A**) Bar plot of all individual almond genotypes generated using STRUCTURE software version 2.3.4 [51]. For the burning phase, 30,000 iterations were set, followed by 1000 repetitions for K values, ranging from 1 to 10, with 10 runs for K. The three reconstructed subpopulations are distinguished by different colors: blue for cluster 1 (**B**), orange for cluster 2 (**C**) and grey for cluster 3 (**D**). Each genotype is represented by a vertical bar that can be portioned into different colored segments indicating its genetic background. Multiple colors show the admixed genetic constitution of some individuals. Cluster assignment was based on a membership threshold set at >0.6.

Structure analysis allowed us to determine the genetic constitution of the Apulian almond population. Genotypes whose membership coefficient (qi) was higher than 0.6 were assigned to a defined cluster, otherwise they were considered to be of admixed ancestry. In accordance with the Evanno criterion [50], a strong signal for K = 3 was obtained (Figure 2), thus indicating three main, clearly genetically distinct groups that mirrored the genetic distance-based clustering, plus one admixed group (Figure 3). The first cluster (blue) grouped together 62 genotypes that mostly corresponded to the G-1A clade; the second cluster (orange) consisted of 48 genotypes that included genotypes of the G-1B clade, while the third cluster (grey) included 45 genotypes belonging to the G-2 clade. Again, all the commercial varieties used as references fell into one single cluster (n.1). Reflecting the phylogenetic tree, the cultivars “Tuono” and “Troito”, as well as “Mollesca di Ruvo”, “Stilla” and “Piangente”, grouped together in cluster 2, thus providing further confirmation of their genetic similarity.

Many cases of admixture (32 in total) were evident within and among gene pools, likely as a result of hybrid origin and allele sharing among these genotypes. As an example, the cultivars “Filippo Ceo” and “Falsa Barese” were assigned to the admixed group in this study, highlighting their different allelic composition, as also confirmed by [35,48]. Nevertheless, contrasting results may emerge depending on the sample dataset of the collection analyzed and the kind of markers used, as in the research works by [17,39], that assumed a common origin for these two cultivars, or else that one could have originated from the other.

The alleles richness of the almond Apulian germplasm, confirmed by the high number of alleles scored, with a relatively congruent number of SSR loci analyzed, reflected the high level of diversification within the Apulian germplasm. This diversification was also verified and confirmed in both the neighbor-joining and structure plot that gathered the genotypes analyzed within clearly distinguishable groups.

### 2.4. Phenotypic Characterization

**Figure 4:**
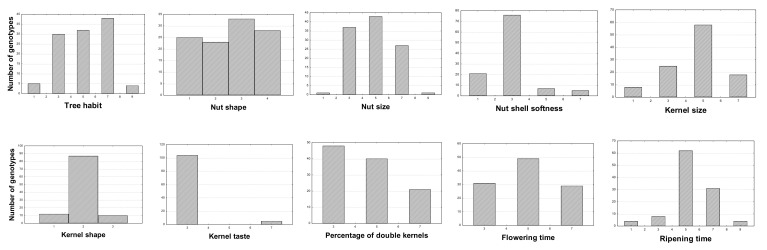
Distribution of the 109 almond genotypes for morphological and phenological traits scored according to GIBA code.

**Figure 5:**
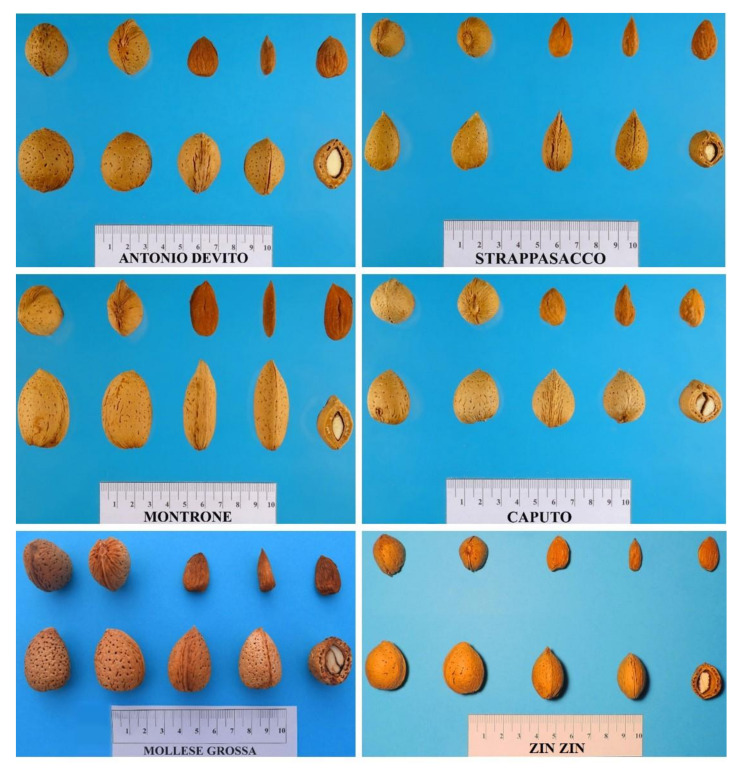
Nut and kernel morphology of some Apulian almond genotypes.

A subset of 109 cultivars was subjected to a detailed phenotypic characterization. Descriptive statistics of the analyzed traits are shown in Table 3, including means, minimum, maximum and coefficient of variation (CV), and in Figure 4, illustrating the distribution of Apulian almonds in terms of the most important agronomic traits. The selected genotypes showed good levels of phenotypic variation as regards all traits, as confirmed by the relatively high coefficient of variation (CV) values (Table 3), as had also been highlighted by [16] in a study of a large population of Apulian almonds. Among the traits measured, the highest level of variation was found for nut ventral suture (CV = 89.70%) while kernel tegument color intensity showed the lowest differences among genotypes (CV = 20.78%). Among technological traits, mean values of nut and kernel size were medium (Table 3 and Figure 5), but as shown in Figure 4, some cultivars exhibited values rated in ranges from very small to very large. Nutshell resistance to cracking resulted hard in most of the population examined; the mean number of double kernels was low-medium; all these results are in agreement with [16]. Flowering time was intermediate in most of the cultivars, but a group of 31 genotypes showed an early blooming time, while another group of 29 genotypes resulted late (Figure 4). Ripening time in most of the cultivars (62) was intermediate, with only few cultivars exhibiting very early (4) and very late (4) ripening times (Figure 4).

Principle component analysis assigned most of the traits to four components which explained 46.1% of the total variation (Table 4). The first two components, which accounted for 27.4% of the total variation, highlighted technological characteristics such as nut size, nut shape, percentage of double kernels in PC1 and nut ventral suture, nutshell softness and kernel size in PC2. The phenological traits showed the highest factor loadings in PC3 (flowering time) and PC4 (ripening time).

**Figure 6:**
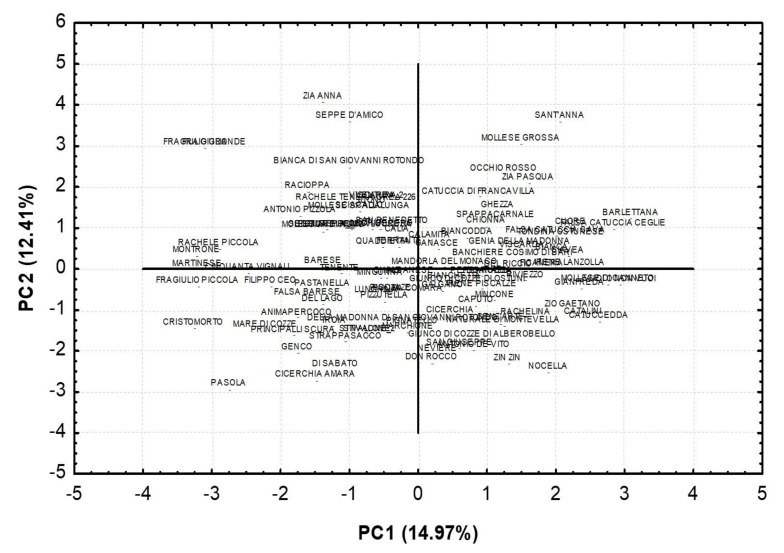
Factor scores of the first two principal components (PCs) for almond genotypes.

Based on the first two components, the PCA plot grouped the almond cultivars according to their technological features resemblance (Figure 6). Proceeding from positive to negative values of PC1, genotypes were characterized by a lower percentage of double kernels and higher nut size. Proceeding from positive to negative values of PC2, cultivars were defined by a lesser nutshell softness and smaller kernel size. The distribution of almond genotypes on the PC1 and PC2 plots confirmed the wide variability of the samples. A similar wide variability for some of the phenotypic traits analyzed was confirmed by other investigations conducted on a different Apulian almond population [9,17].

**Table 3:** List of morphological and phenological traits detected in the almond collection and their descriptive statistics.

Trait	Mean	Expression Level	Minimum	Maximum	Coefficient of Variation (CV %)
Tree habit	5.11	medium	1.00	9.00	38.35
Tree vigor	6.05	medium-strong	1.00	9.00	25.40
Color of petals	1.27	white	1.00	3.00	43.85
Leaf blade color	5.50	green	3.00	7.00	22.78
Nut size	4.82	medium	1.00	9.00	33.69
Nut shape (side view)	2.59	round-oblong	1.00	4.00	42.80
Nutshell color intensity	4.45	light-medium	1.00	9.00	38.62
Nutshell incision (pores)	2.27	medium porous	1.00	4.00	33.24
Nut ventral suture	1.73	firmly closed	1.00	5.00	89.70
Nutshell softness	2.93	hard	1.00	7.00	45.49
Kernel shape	1.98	elliptic	1.00	3.00	22.76
Kernel size	4.58	medium	1.00	7.00	35.21
Kernel tegument color intensity	5.27	medium	3.00	7.00	20.78
Kernel taste	3.18	sweet	3.00	7.00	26.41
Percentage of double kernels	4.50	low-medium	3.00	7.00	33.72
Flowering time	4.96	intermediate	3.00	7.00	30.03
Ripening time	5.42	intermediate	1.00	9.00	28.87

**Table 4:** Eigenvalues and proportion of total variability for the first eight principal components from PCA analysis of the almond genotypes studied.

Traits	PC1	PC2	PC3	PC4
Tree habit	0.09	−0.13	−0.50	0.07
Tree vigor	−0.09	−0.07	0.22	0.61
Color of petals	−0.35	−0.47	0.30	−0.25
Leaf blade color	0.31	−0.07	−0.25	−0.26
Nut size	−0.63	0.49	−0.25	0.16
Nut shape (side view)	−0.73	0.08	−0.20	0.06
Nutshell color intensity	−0.43	−0.27	0.44	−0.05
Nutshell incision (pores)	−0.33	0.26	0.16	−0.09
Nut ventral suture	−0.21	0.50	0.29	−0.23
Nutshell softness	0.30	0.54	−0.10	−0.41
Kernel shape	−0.50	−0.05	−0.40	−0.25
Kernel size	−0.23	0.61	−0.24	0.43
Kernel tegument color intensity	−0.35	0.25	0.49	0.15
Kernel taste	−0.16	−0.27	0.33	0.05
Percentage of double kernels	0.56	0.31	0.08	0.38
Flowering time	−0.41	−0.29	−0.47	−0.07
Ripening time	−0.02	−0.49	−0.26	0.52
*% of Variance*	*14.97*	*12.41*	*10.14*	*8.56*

**Figure 7:**
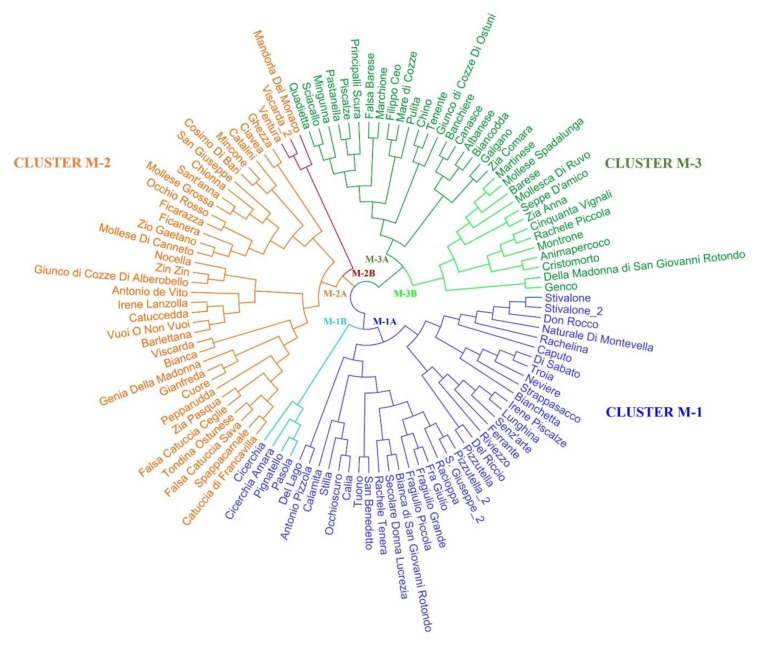
Morphological dendrogram generated by the neighbor-joining clustering method using all 17 phenotypic descriptors on a subset of 109 almond genotypes.

The neighbor-joining clustering method utilizing all the morphological and phenological data again subdivided the Apulian almond population into three main clades with comparable dimensions (Figure 7). Indeed, 39 cultivars belonged to cluster M-1, 37 were included in cluster M-2, and 33 were included in cluster M-3, the least abundant. Moreover, within each cluster it was possible to identify at least two subgroups. The subclusters M-1A and M-3A mostly included almonds with large size nuts and kernels, but the greater nut size did not always correspond to the greater kernel size. Genotypes showing the highest values of shell hardness fell into subgroups M-2A and M-3A. The main cluster M-2 grouped all genotypes with the highest percentage of double kernels. Almonds with the most bitter kernel taste were included in subcluster M-1B.

The observed morphological variability mostly mirrored the results of the molecular characterization obtained with SSR markers, especially the M-1 clade, that was almost exclusively composed of genotypes included in the G-1 clade. Moreover, most of the genotypes with a high genetic similarity according to their LRM values were once again found to belong to the same branches of the morphological tree. For example, the high genetic similarity between the cultivars “Stilla” and “Tuono” (LRM = 0.5), “Bianchetta” and “Lunghina” (LRM = 0.49), “Del lago” and “Rachele tenera” (LRM = 0.48) also emerged from the phenotypic analysis, these genotypes being included in the same morphological subclusters.

However, some exceptions and discrepancies between the morphological and the genetic analysis emerged, as also reported by [47], which highlighted how some traits, such as kernel oil composition, for example, could be strongly affected by the season, location and climate of the tree growing areas, despite being primarily genotype-dependent. Thus, as expected, also in our study, some genotypes sharing most of the genetic background for the considered microsatellite loci resulted phenotypically rather distant, probably due to their different growing environments. This is, for example, the case of “Mollesca di Ruvo” versus “Tuono” and “Stilla”, that shared high genetic similarity, but in the morphological analysis they fell into different clades. On the contrary, two cases of synonymies, that were clearly genetically resolved by SSR analysis, were found to be close to each other in terms of phenological and morphological characterization, thus explaining the attribution of the same name to these genotypes (this is the case of “Pizzutella” and “Pizzutella_2” and of “Stivalone” and “Stivalone_2”) (Figure 7).

## 3. Materials and Methods

### 3.1. Plant Material

A total of 187 Apulian almond genotypes from different locations was considered in this study. Among them, 41 belonged to the ex situ collection of CRSFA Institute (Centro di Ricerca, Sperimentazione e Formazione in Agricoltura “Basile Caramia”) located in Locorotondo (Bari); 23 derived from the Di.S.S.P.A (Department of Soil, Plant and Food Sciences) germplasm collection of the University of Bari, located in Valenzano (Bari); 43 were sampled in the experimental field of the CREA Institute (Council for Agricultural Research and Economics-Agriculture and Environment Research Centre) located in Bitetto (Bari), and 74 were kindly provided by local farmers from different areas in the Apulia region. In addition, 6 foreign commercial varieties provided by CRSFA were added as reference.

### 3.2. DNA Extraction and SSR Analysis

Young leaves were collected from each plant tree and stored at −20 °C until the use. Genomic DNA was extracted according to [52]. The quantity and quality of extracted DNA were evaluated by spectrophotometric measurement of absorbance ratios and by electrophoresis on 0.8% Certified Molecular Biology Agarose gel (Bio-Rad Laboratories, Hercules, CA, USA) in 1 X TBE buffer (1 M Trizma, 1 M Boric Acid, 20 mM EDTA, pH 8.3). All concentrations were normalized to 50 ng/μL with 0.1 X TE buffer (10 mM Tris-HCl pH 8.0 and 1 mM EDTA).

A set of 18 nuclear SSR was used to assess the genetic variability of the almond collection. For each SSR, information about the type of locus, repeat motif, annealing temperature, linkage group and species in which they were isolated, are reported in Table 5. Amplification reactions were performed in a C1000TM Thermal Cycler (Bio-Rad Laboratories, Hercules, CA, USA) in a final volume of 25 µL, containing 1 X Dream Taq Buffer, 0.04 M dNTP mix, 0.25 µM primer mix, 0.03 U/µL Dream Taq and 50 ng of genomic DNA. All reagents were provided by Thermo Fisher Scientific^®^ (Waltham, MA, USA) and the forward primer of each couple was labelled with one of the following dyes: 6FAM^™^, NED^™^, VIC^®^ and PET^™^. In order to optimize the reaction times, the same touchdown protocol was applied for all the selected SSRs, consisting of an initial denaturation at 94 °C for 5 min; 10 cycles of 94 °C for 30 s, followed by an annealing step at 55 °C for 45 s, and 1 min at 72 °C; then, 25 cycles at 94 °C for 30 s, 50 °C for 45 s, and 1 min at 72 °C; a final extension was run at 72 °C for 30 min.

PCR products were prepared as described by [52], separated by the automatic capillary sequencer ABI PRISM 3100 Avant Genetic Analyzer (Applied Biosystems, Foster City, CA, USA) with the internal molecular weight standard GeneScan Liz 600 dye (Applied Biosystems, Foster City, CA, USA) and finally genotyped by GeneMapper software v.5.0 (Applied Biosystems, Foster City, CA, USA).

**Table 5:** List of microsatellites used in this study. Locus type, species of origin (*Prunus* spp), almond linkage group (LG), repeat motif and annealing temperature (Ta in °C) are specified.

SSR Locus	Type	Species *	Almond LG *	Motif	Ta	Reference
BPPCT001	SSR	*P. persica*	2	(GA)_27_	57	[53]
BPPCT007	SSR	*P. persica*	3	(AG)_22_(CG)_2_(AG)_4_	57	[53]
BPPCT010	SSR	*P. persica*	4	(AG)_4_GG(AG)_10_	57	[53]
BPPCT014	SSR	*P. persica*	5	(AG)_23_	57	[53]
BPPCT025	SSR	*P. persica*	6	(GA)_29_	57	[53]
CPDCT025	SSR	*P. dulcis*	3	(CT)_10_	62	[54]
CPDCT042	SSR	*P. dulcis*	1	(GA)_27_	62	[55]
CPDCT045	SSR	*P. dulcis*	4	(GA)_16_	62	[55]
CPPCT006	SSR	*P. persica*	8	(CT)_16_	59	[56]
CPPCT033	SSR	*P. persica*	7	(CT)_16_	50	[56]
CPSCT012	SSR	*P. salicina*	6	GA	62	[54]
CPSCT018	SSR	*P. salicina*	8	(CA)_5_(CT)_20_	52	[54]
EPPCU5176	EST-SSR	*P. persica*	7	-	55	[57]
PCHGMS1	SSR	*P. persica*	2	(AC)_12_(AT)_6_	60	[58]
UDP96003	SSR	*P. persica*	4	(CT)_11_(CA)_28_	57	[59]
UDP96005	SSR	*P. persica*	1	(AC)_16_TG(CT)_2_CA(CT)_11_	57	[59]
UDP98409	SSR	*P. persica*	8	(AG)_19_	57	[59]
UDP98412	SSR	*P. persica*	6	(AG)_28_	57	[60]

*: According to information reported in the Genome Database of Rosaceae (GDR, http://www.rosaceae.org accessed on 6 September 2021); -: not available.

### 3.3. Genetic Diversity and Population Structure Analysis

For each SSR marker, the following statistical parameters were calculated using GenALEx software v. 6.51b2 (http://biology-assets.anu.edu.au/GenAlEx accessed on 15 September 2021) [61]: number of alleles (Na), effective number of alleles (Ne), Shannon’s information index (I) [62], observed (Ho) and expected (He) heterozygosity, and fixation index (F) [63]. GenALEx was also used to estimate the number of private alleles [64] and the marker-based relatedness (LRM) in order to infer the degree of relatedness of pairs of individuals [65]. Cervus v. 2.0 allowed us to calculate the polymorphic information content (PIC) and the frequency of null alleles (F-Null).

Genetic distance between the almond genotypes was calculated using the simple matching dissimilarity index. A weighted neighbor-joining tree [66] was computed using the Dissimilarity Analysis and Representation for Windows (Darwin5) (http://darwin.cirad.fr accessed on 16 September 2021) software version 6.0.010. The robustness of branches was tested using 1000 bootstraps [67]. In addition, a model-based Bayesian analysis was performed to evaluate the genetic structure of the collection and to identify putative admixed individuals using STRUCTURE software version 2.3.4 [51], which was run from the command line using the admixture model, a burn-in period length of 50,000, and 50,000 Markov-Chain Monte Carlo (MCMC) iterations after burn-in. Ten independent runs were performed for each K from K = 1 to K = 10. The best number of K was chosen with the DeltaK method by running the STRUCTURE HARVESTER software [68].

### 3.4. Phenotypic Characterization

During the years 2014 and 2015, for each genotype, 15 morphological traits and 2 phenological traits (Table 6) were analyzed and coded according to the descriptors list for Almond of the GIBA (Guidelines for the conservation and characterization of plant, animal, and microbial biodiversity of interest to agriculture, published by the Italian Ministry of Agricultural, Food and Forestry Policies) (www.reterurale.it/flex/cm/pages/ServeBLOB.php/L/IT/IDPagina/9580 accessed on 4 October 2021). Each year, at ripening time, a sample of 100 hulled and dried fruits was taken for each genotype to study nut and kernel traits.

**Table 6:** List of phenotypic traits selected for this study.

TRAIT	
TREE	Habit
	Vigor
LEAF	Blade color
FLOWER	Petal color
NUT	Size
	Shape (side view)
	Shell color intensity
	Shell softness
	Shell incision (pores)
	Ventral suture
KERNEL	Size
	Shape
	Taste
	Tegument color intensity
	Percentage of double kernels
PHENOLOGY	Flowering time
	Ripening time

### 3.5. Statistical Analysis

Mean, minimum, maximum and coefficient of variation (CV) of coded values of traits were calculated for a representative subset of 109 cultivar. The distribution of the selected genotypes for each trait was represented in frequency histograms, based on the mean of coded values of the two years of the study. Relationships among genotypes were investigated by multivariate analysis of variance (principal component analysis, PCA) STATISTICA [69].

## 4. Conclusions

The present study made it possible to explore some aspects of almond biodiversity in Apulia region, by highlighting the hypothetical composition of the autochthonous germplasm. The combination of molecular and morphological data was proven once again to be the best choice in order to characterize the available plant resources. Microsatellites and morphological markers were successfully used to assay the genetic diversity of the Apulian almond collection and underlined a high level of diversification. All the genotypes examined were unequivocally identified, and some cases of misnaming and/or homonymy have been clarified. In particular, this aspect is extremely important and may be a prerequisite in the process of nursery material certification and official registration.

Alongside the detailed characterization of the retrieved trees, the propagation, and the subsequent cultivation of some of them in appropriate collection fields could not only safeguard several genotypes, protecting them from extinction, and preserve the already known cultivars as a source of intravarietal diversity, but also allow planning of the evaluation of almonds in terms of yields and quality. The good adaption of Apulian almonds to the regional environmental conditions makes them an interesting bioresource that could be extremely useful for breeding programs and for the selection of promising, highly productive candidates that may be resilient to the climate changes underway. Moreover, the rediscovery and preservation of indigenous and putatively endangered almond cultivars, especially in marginal areas, may also have important repercussions and benefits on the local economy, further enhancing the value of the Apulian almond germplasm.

Thus, the extensive genetic variability of the Apulian almond germplasm that emerged in this study indicates that these materials are an important source of genes for almond improvement and valorization. Our work offers information that may stimulate further breeding efforts to enhance the sustainability of this crop.

## Data Availability

All data of molecular and morphological characterizations have been collected in an integrated database with the regional GIS portal, accessible on the Apulia region website (misura 10.2.1: www.psr.regione.puglia.it accessed on 18 May 2021) on request. Historical information and detailed pictures of almond genotypes are reported in the “Atlante dei frutti antichi di Puglia”, available at the link: http://www.fruttiantichipuglia.it/atlante-dei-frutti-antichi-di-puglia/ accessed on 18 May 2021.
